# Evaluating Case-Based Learning to Enhance Dental Student Performance in Cavity Preparation: A Pilot Study

**DOI:** 10.7759/cureus.69222

**Published:** 2024-09-11

**Authors:** Osama Khattak, Najem Ghanem Alruwaili, Layan Mohammed M Alarjan, Aljowharah Ali Alsattam, Rabia Anis, Azhar Iqbal, Farooq Ahmad Chaudhary

**Affiliations:** 1 Department of Restorative Dentistry, Jouf University, Sakaka, SAU; 2 College of Dentistry, Jouf University, Sakaka, SAU; 3 Department of Health Professions Education, Isra Dental College, Isra University, Hyderabad, PAK; 4 School of Dentistry, Shaheed Zulfiqar Ali Bhutto Medical University, Islamabad, PAK

**Keywords:** case-based learning, curriculum, dental education, dental students learning, operative dentistry

## Abstract

Background

The transition from theoretical knowledge to clinical competency in operative dentistry is an ongoing challenge in dental education. Undergraduate students often face a significant gap between understanding theory and applying practical skills for diagnosing and preparing cavities, which leads to decreased confidence, increased stress, and potential errors in clinical practice. Case-based learning (CBL), an active learning strategy that emphasizes real-world scenarios, has shown promise for improving clinical skills and knowledge retention in other healthcare disciplines. This pilot study explored the potential of CBL to address the theory-practice gap and enhance confidence among undergraduate dental students in performing cavity preparations.

Methods

This pilot study utilized a pre and post-intervention design and involved 30 second-year dental students. Participants completed confidence surveys at three points: before CBL activity, immediately after CBL activity, and following their first clinical cavity preparation procedure. The CBL activity consisted of two components: interactive case discussions and hands-on laboratory exercises designed to simulate real-world dental procedures. The interactive discussions focused on diagnostic and treatment planning while laboratory exercises provided practical experience in cavity preparation. Data were analyzed using repeated-measures analysis of variance (ANOVA) to assess the changes in confidence levels across the three survey points.

Results

CBL significantly increased students’ confidence across multiple dimensions (p < 0.05). Confidence in modifying cavity preparations increased from a mean of 2.91 (SD = 0.292) before CBL to 5.12 (SD = 0.331) post-CBL and further to 5.52 (SD = 0.556) after clinical practice. Similarly, confidence in understanding the basic concepts of caries removal improved from 4.82 (SD = 0.528) pre-CBL to 5.45 (SD = 0.564) post-CBL, and to 5.61 (SD = 0.556) post-clinic. However, confidence in performing cavity preparations without faculty guidance remained relatively low, increasing from a baseline mean of 2.67 (SD = 0.478) to 2.91 (SD = 0.384) post-CBL and 3.64 (SD = 0.603) post-clinic.

Conclusion

Incorporating CBL into the curriculum can significantly improve undergraduate students’ confidence in performing cavity preparation. This approach may help bridge the theory-practice gap and better prepare students for the clinical environment. This pilot study provides promising initial data, and further research is needed to determine the long-term impact of CBL and the optimal implementation strategies.

## Introduction

Operative dentistry is a fundamental branch of dentistry that focuses on the diagnosis, prevention, treatment, and prognosis of diseases or traumas affecting dentition. It encompasses a significant portion of the dental curriculum, with operative clinical procedures being among the first procedures undertaken by students [1]. The curriculum typically includes theoretical instruction, preclinical laboratory sessions, and clinical training. The preclinical laboratory component aims to enhance students' psychomotor skills through optimal cavity preparation techniques for typodont teeth [1,2].

Theoretical teaching alone is insufficient, as advancements in restorative materials have led to minimally invasive procedures [3]. Additionally, advanced cariology has influenced treatment plans [4]. Therefore, it is necessary to integrate case-based teaching methodologies into the current educational system. Conservative tooth preparation plans can be better implemented through increased case-based practical training [5].

In-depth preclinical simulation exercises for typodont teeth do not address all transitional clinical challenges. Clinical tooth preparation is more demanding and may involve complications such as chronic disease management, genetic disorders, and caries extension [6]. The disparity between predoctoral education and clinical experience creates a gap between theory and practice, leading to insufficient knowledge and an imperfect learning environment [7]. This contributes to decreased confidence and increased stress among students, particularly during their early clinical years [8]. Although experience can bridge this gap, immediate rectification is required. Increased clinical competence correlates with increased confidence [9]. Therefore, incorporating more case-based preclinical practices into the curriculum can enhance student training and confidence.

In many North American dental schools, pre-doctoral students start clinical operative procedures only after completing pre-clinical formative exercises and competency exams on typodont teeth [10]. Various institutions employ different case-based methodologies and preclinical exercises to address these transitional challenges. Some researchers have adopted haptic virtual reality (VR) simulation systems, 3D-printed teeth with varying textures, and real-life extracted teeth [11,12]. Although commendable, these tools may not be accessible, cost-effective, or time-efficient. Thus, an effective, easily implemented intervention to bridge the theory-practice gap is necessary [13].

Case-based learning (CBL) has proven to be a highly effective teaching approach in healthcare education [14]. CBL offers lifelong learning benefits, enhances skill application, and improves knowledge retention [15]. Additionally, CBL boosts confidence by enhancing communication and collaboration skills [16,17], thereby improving clinical treatment planning at multiple levels [18]. As an active learning methodology, CBL promotes self-study, problem-solving, and critical thinking [19]. The Commission on Dental Accreditation (CODA) emphasizes critical thinking among the competencies required for dental graduates. CBL effectively enhances students' critical thinking and decision-making abilities, surpassing traditional lecture-based approaches, and fulfilling the competency requirement [20].

Critical thinking ability and self-confidence are positively correlated [21]. Therefore, CBL activities not only promote lifelong skills but also increase students' confidence. CBL has been employed by healthcare programs worldwide to bridge the gap between theory and practice, thereby enhancing students’ clinical reasoning and critical thinking abilities [22].

Considering the above-mentioned points, a study was conducted to assess the effectiveness of case-based activities in bridging the gap between theoretical knowledge and practical skills by evaluating the confidence levels of students while performing operative procedures in clinical settings. The null hypothesis suggested that case-based activities would not affect students' confidence level in conducting operative procedures.

## Materials and methods

This study was approved by the Local Committee of Bioethics (LCBE), Jouf University, Saudi Arabia (Reference no. 2-11-44). Thirty students who had completed the second year of the dental program participated in the study. The sample size for this study was determined based on the number of final-year dental students available during the study period. Given that this study was designed as a pilot to explore the feasibility of the case-based learning (CBL) approach, the focus was on gathering preliminary data rather than achieving a specific power calculation. The sample size reflects the entire cohort of students who were available and willing to participate in the study. Future research will include larger sample sizes to enable more rigorous statistical analyses and more definitive conclusions. A case-based study was incorporated into the curriculum to encourage student participation, with the completion of surveys required. The survey was conducted in three separate stages. The initial survey, known as the "post-course survey," was conducted following the completion of the main theoretical operative course. The results of this survey indicated the level of confidence that students had in performing a clinical procedure immediately following their theoretical course.

This study was designed as a pilot to explore the feasibility and initial impact of case-based learning (CBL) on dental students' confidence and performance. Due to the exploratory nature of the study and resource constraints, a control group was not included. The primary aim was to assess the potential benefits of CBL in a real-world educational setting, rather than to conduct a comparative analysis against traditional teaching methods.

Following the theoretical course, students participated in a case-based activity, after which a post-activity survey was administered. The third survey, referred to as the "follow-up survey," was conducted after the students completed their first clinical procedure following the second survey. All surveys had identical questions and consisted of eight items that asked students to rate their confidence level at different stages of preparing a cavity in the clinic using a five-point Likert scale ranging from 1 to 5 (Appendices).

The questionnaire used in this study was developed by our research team to assess student confidence levels in performing cavity preparation before and after the case-based learning intervention. The questionnaire underwent an internal validation process, including expert review by senior faculty members in the Department of Dental Education and pilot testing with a small group of students (n=5). The reliability of the questionnaire was confirmed by calculating Cronbach's alpha, which yielded a value of 0.80, indicating acceptable internal consistency. Based on the pilot feedback, minor revisions were made for clarity and comprehensiveness.

To gather more suggestions for improvement, a feedback survey was administered to both teachers and students at the end. This survey included open-ended subjective questions. Surveys were generally distributed and collected as hard copies, but some were conducted online for convenience.

**Figure 1:**
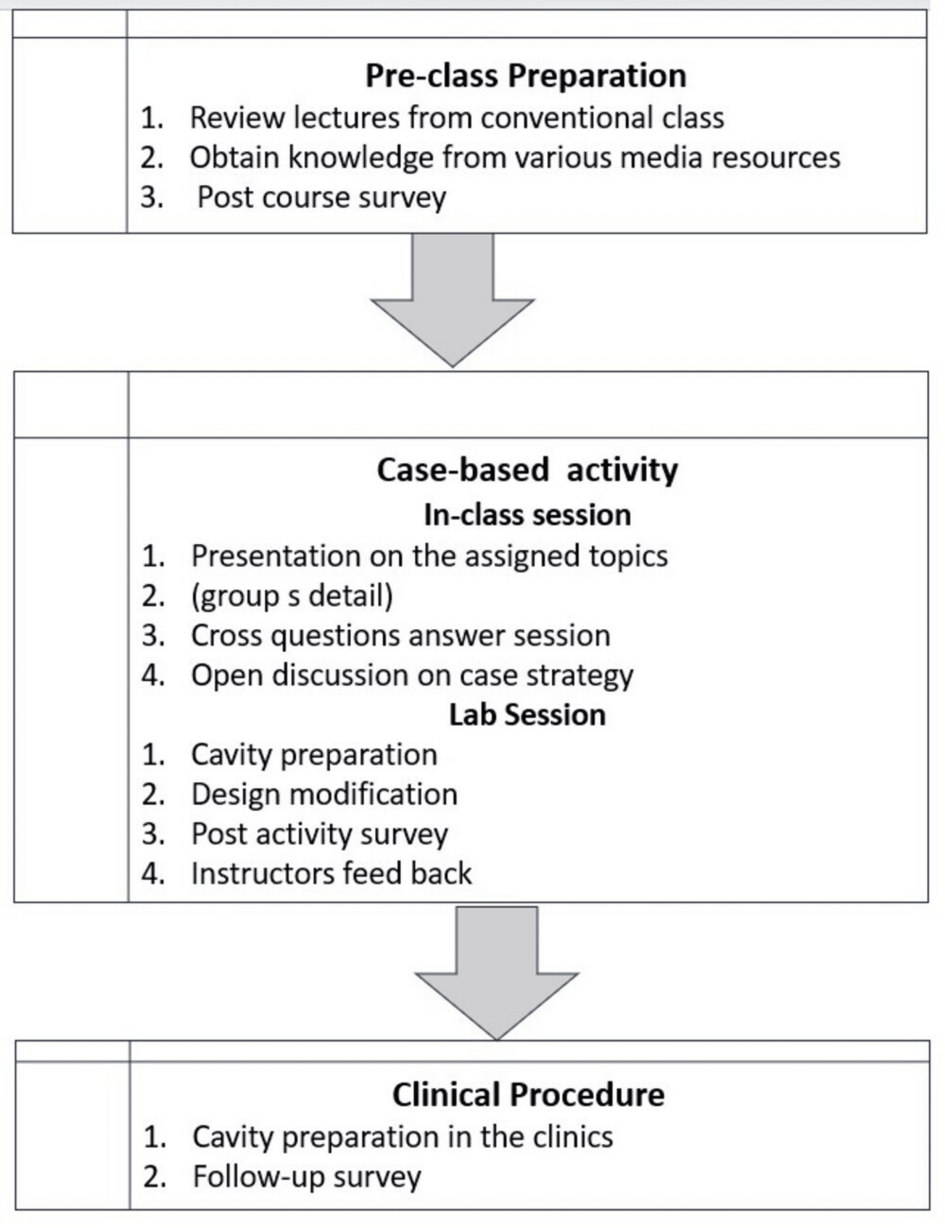
Flowchart illustrating the sequence of activities in the case-based learning (CBL) approach The figure details the diagnostic process, material selection, procedural steps, and feedback sessions, providing a comprehensive overview of how the CBL framework was implemented in the study.

The case-based activity consisted of two components: a team-based interactive session in class, followed by a lab session. Before engaging in the case-based activity, students were instructed to revisit the operative concepts they had learned in lectures and were encouraged to acquire additional knowledge from media sources. To ensure their preparedness, a short quiz was conducted before the activity session. Figure 1 illustrates the transition between the various stages and steps of an activity.

Students were divided into groups of four to five, each assigned a distinct classification of tooth preparation (GV Black Class I, II, III, IV, and V). Each group was provided with multiple clinical scenarios presented in folders containing photographs and radiographs of lesions. The students were required to develop and present a step-by-step procedure to approach each lesion, following the outline in Table 1. This presentation was followed by intergroup discussions and cross-questioning sessions, guided by teachers, covering topics such as suggested restoration materials and access approaches to cavity formation.

**Table 1:** Proposed procedure outline

Procedure Outline	
1	Diagnose the lesions
2	Required material and rationale for particular material selection
3	Required equipment
4	Isolation steps
5	Steps for anesthesia
6	Method used in the detection of carious lesions
7	Preparation design selection (reasons for selecting invasive vs. extensive)
8	Pulp protection considerations
9	Steps for restoration placement

The in-class session was immediately followed by a lab session in preclinical laboratories. In this session, students implemented their proposed preparation plans on simulated typodont teeth with carious lesions, without faculty support or instructional guidance. They independently prepared the cavities based on their knowledge from lectures and discussions. This exercise was followed by a post-activity survey to evaluate the effectiveness of the interactive class activity. Instructors then evaluated the teeth and provided feedback to the students.

After a few months, students performed their first clinical operative procedure, informed by the feedback from the lab session. This was followed by the third (follow-up) survey. An additional feedback survey was administered to both students and faculty, containing both qualitative and quantitative questions, to invite suggestions for improvement. Key questions included the type of tooth used in the lab, the number and type of cases performed, and the recorded approach.

For data analysis, repeated measures ANOVA was employed to assess changes in student confidence levels across three time points: before the CBL activity, immediately after the CBL activity, and following the students' first clinical cavity preparation procedure. The repeated measures ANOVA was selected because it allows for the comparison of means within the same group over multiple time points while accounting for the correlation between repeated measurements from the same subjects. The level of statistical significance was set at α = 0.05. All statistical analyses were performed using SPSS Version 26 (IBM Corp., Armonk, NY, US).

## Results

The study achieved a 100% response rate across all three surveys, involving a sample size of 30 students. The results demonstrated a significant increase in confidence levels across various parameters after the completion of a case-based learning (CBL) activity and the initial operative procedures during teaching practice. Confidence in modifying cavity preparation saw a substantial rise, with the mean score increasing from 2.91 (SD = 0.292) before CBL to 5.12 (SD = 0.331) post-CBL, and further improving to 5.52 (SD = 0.556) after clinical practice (Table 2). Additionally, the understanding of basic concepts of caries removal improved from a mean score of 4.82 (SD = 0.528) pre-CBL to 5.45 (SD = 0.564) post-CBL, reaching 5.61 (SD = 0.556) after clinical practice (Table 2). However, the confidence in performing cavity preparations without faculty guidance, while showing improvement, remained relatively low. The mean score increased from 2.67 (SD = 0.478) at baseline to 2.91 (SD = 0.384) post-CBL, and further to 3.64 (SD = 0.603) after clinical practice (Table 2).

**Table 2:** Survey statement results Test statistic (F-value): The F-values have been calculated using repeated measures ANOVA for each survey statement across the three time points. ANOVA: analysis of variance

Survey Statement	Post-Course Survey Mean (SE)	Post-Activity Survey Mean (SE)	Follow-Up Survey Mean (SE)	Test Statistic (F-Value)	P-Value
I feel comfortable modifying my cavity preparation	2.91 (0.292)	5.12 (0.331)	5.52 (0.556)	F= 6.45	< .05
I mentally feel comfortable enough to design a cavity preparation	2.85 (0.364)	5.21 (0.415)	5.24 (4.35)	F=5.87	< .05
I will not require faculty support during my first clinical procedure	2.67 (0.478)	2.91 (0.384)	3.64 (0.603)	F=4.12	< .05
I am confident to evaluate my procedure	4.85 (0.442)	5.18 (0.392)	5.06 (0.242)	F=3.98	.002
I understand the basic concepts of caries removal	4.82 (0.528)	5.45 (0.564)	5.61 (0.556)	F=6.33	< .05
I have clear concepts of cavity preparation	5.15 (0.442)	5.42 (0.614)	5.52 (0.508)	F=4.67	0.017
I can comfortably select the appropriate required bur	4.48 (0.508)	5.12 (0.331)	5.45 (0.506)	F=5.21	< .05
I am confident of my motor skills	4.15 (0.755)	5.03 (0.394)	5.18 (0.392)	F=4.92	< .05

After the preclinical lab activity session, the faculty provided feedback, and the cavity preparation approaches of the students were recorded and analyzed. The results indicated that only 15% of the students performed conventional material-based tooth preparations while 85% opted for a minimally invasive design, which included separating the mesial pit and distal occlusal tooth preparation (Table 3).

**Table 3:** Results of the responses of the feedback survey

Survey Question	Response Options	Response Percentage
Approach followed by students	Conventional	15%
	Conservative	85%
Type of tooth to be used in lab activities	Want real tooth	63%
	Want typodont tooth	37%
Want more discussion sessions	Want more sessions	68%
	Are satisfied	32%
Want more case-based activities	Want more sessions	78%
	Are satisfied	22%
Level of teacher interaction	More intervention	75%
	Are satisfied	25%

The students' feedback regarding the case-based activities was overwhelmingly positive, with some of their comments highlighted in Table 4. The analysis of the feedback survey suggested that students desired an increase in faculty involvement during clinical procedures. Additionally, they expressed a preference for working on real or haptic teeth rather than typodont teeth.

**Table 4:** Summary of students' qualitative feedback following the case-based learning (CBL) activity The table highlights key comments regarding the effectiveness of the CBL sessions, suggestions for improvement, and the perceived impact on their confidence and clinical skills.

Students’ Feedback
"Helped me out a lot and looking forward to having more."
"I think we should do more of such practical classes rather than ideal preparations."
"It increased my cavity preparation knowledge and helped me know how far to extend the cavity in my clinical practice."
"The case-based activity discussion portion helped a lot in improving my cavity preparation plan. Such discussions should be part of normal classes also."
"It will be more helpful if the cavities in the typodont tooth had varied textures. This would allow us to use different tools."
"The experience can be further improved by bringing in actual teeth."
"It would be a good idea to drill on actual teeth, but I know it will not be that easy to manage."

The survey also revealed that students were in favor of incorporating multiple case-based sessions into their curriculum, as they felt these sessions significantly enhanced their experience and confidence. Furthermore, the students recommended that multiple discussion sessions be organized, with different groups assigned to various cases. This approach would provide them with practical support in a range of clinical scenarios.

## Discussion

The outcomes suggest the efficacy of case-based activities. A substantial increase in students' self-assurance, particularly in their ability to execute initial preclinical surgical procedures, has been observed. Thus, the null hypothesis was rejected, and an increase in confidence levels was observed for all parameters. The results were suggestive of improving the confidence level and comfort while approaching cavity lesions.

Although there was a slight improvement to some degree in the follow-up survey in comparison with the post-activity survey, this implied that the students' readiness increased after performing the procedure on a real tooth. This also coincided with the students' descriptive feedback, in which about 63% of the students suggested that their lab experience could be enhanced through real teeth. The students indicated that they had low levels of confidence when adjusting the design of the cavity preparation for an actual tooth. This issue can be addressed by introducing better-textured typodont teeth and haptic teeth in the laboratory section of the activity session. This experience can be further enhanced by allowing the students to carry out lab practice on real teeth [23].

Confidence levels were relatively lower after routine teaching practice than after the post-activity session. The mean values of the post-activity and follow-up surveys showed a substantial increase compared with the post-course survey. It can be inferred that students benefited from the activity and entered their clinics with more confidence and preparedness than they did with the conventional teaching methodology. After completing the activity, students indicated a high level of confidence in their understanding of the principles involved in caries removal and the basics of tooth preparation using both handheld and rotary instruments. This finding suggests that the activity led to an increase in students' confidence in both their knowledge and practical hand skills. Thus, it can be deduced that the activity made students stand ahead of their usual expected standards and gave them more confidence in the application of their knowledge in practical life.

The feedback survey analysis revealed that 68% of students wanted more discussion sessions to be held, and 78% desired more case-based activity sessions to be included in their curriculum. Thus, engaging the students in such activities and integrating lessons that stimulate conceptual clinical knowledge helps them better understand multiple clinical scenarios [24]. Students were able to solve and plan their treatment approaches more effectively. Discussions in the case-based activity session allowed the students to incorporate various complications into their treatment plans. This triggered their thinking capacity, broadening their horizons of treatment plans. This contributes to better readiness for clinical procedures. Similar studies conducted in other dental and medical schools where such efforts were initiated revealed that lessons integrated with eliciting conceptual clinical understanding proved to be very effective and helpful in clinical procedures and practices [25].

The examination of the parameter "carrying out their initial clinical procedure with minimal guidance from faculty" revealed that students demonstrated a lesser degree of confidence. Students reported scores less than neutral for this parameter in all three stages. The students further indicated their disapproval in the feedback survey where 75% of the students disapproved of minimal teacher interaction during labs. The activity session enhanced students' ability to apply theoretical knowledge to practical clinical procedures and prepared them for the transition from theory to practice. However, students lacked confidence in applying their knowledge and skills to clinical procedures without faculty guidance. The activity on its own was not enough to resolve the problem of the student's confidence level in performing their initial surgical procedures with only limited guidance [26,27]. This problem can be resolved by allowing the student to independently devise the preparation plan and provide more teacher guidance during the implementation of the preparation during lab sessions. This will reinforce the students' treatment plan and approach towards the lesion, thereby enhancing their confidence levels. Most US dental schools view pre-clinical competency assessments as sufficient indicators of students' preparedness for transitioning to patient treatment and care in clinical settings despite any inconsistencies [28].

Nevertheless, a thorough analysis uncovered the presence of a theory-practice discrepancy. It can be inferred that although students may possess adequate conceptual comprehension and proficiency in performing preclinical procedures, they may lack confidence in applying their knowledge and skills in practical settings such as in a clinical environment. Thus, the activity was insufficient to raise confidence levels sufficiently high to completely overcome the theory-practice gap. The results of this study are consistent with those of another study that found that students' conceptual knowledge is not an effective measure of their ability to perform clinical procedures with confidence [7]. An in-depth analysis of the feedback survey also suggested that this may be because the students never worked on real teeth. The student suggestions analysis indicated that 63% of the students wanted to work on real teeth to enhance their experience. Fear of working on a real tooth may also be a reason for the lack of confidence. This is further to be investigated. In a previous study, students lacked adequate preparation for their initial prosthodontic procedure in a clinical setting environment [7].

While the study shows a significant increase in self-reported confidence among students, it is important to acknowledge the inherent limitations of self-reported data. Confidence levels reported by students may not directly correlate with actual competency or skill levels, as self-assessment can be influenced by various biases, including social desirability bias and overconfidence. This potential disconnect between perceived confidence and actual performance suggests the need for future research to incorporate objective measures of clinical performance, such as faculty assessments or practical exams, alongside self-reported data. Additionally, longer-term follow-ups would be valuable in assessing whether the increased confidence observed immediately after the intervention translates into sustained improvements in clinical outcomes over time [3,5].

The qualitative feedback provided by students further supports the effectiveness of the CBL sessions. Students particularly valued the hands-on and interactive components, which they felt significantly contributed to their understanding and confidence. Many participants expressed a desire for more frequent integration of such activities into the curriculum, highlighting the perceived benefits of CBL in enhancing their learning experience. This feedback aligns with existing literature that emphasizes the effectiveness of active learning strategies, such as CBL, in promoting deep learning and skill acquisition compared to traditional lecture-based approaches [10-12].

Despite the promising findings, several limitations of the study should be acknowledged. First, the reliance on self-reported data as a primary measure of outcome introduces potential bias, as discussed earlier. Second, the study lacked a control group, which limits the ability to make definitive causal inferences about the effectiveness of CBL compared to other instructional methods. Additionally, the study was conducted within a single institution, which may affect the generalizability of the results. Future research should address these limitations by incorporating control groups, objective performance measures, and larger, more diverse sample sizes across multiple institutions. These steps will help validate the findings and provide a more comprehensive understanding of the impact of CBL on dental education [6].

In general, the feedback survey aligned with the results of the three-stage surveys. The students generally provided positive feedback regarding both interactive classes and laboratory exercises, with a few exceptions. They considered it helpful in boosting their confidence and suggested having more such activities embedded in their curriculum. The analysis suggested that they valued the activity sessions and expected more sessions with suggestive improvements.

The modification in the curriculum by incorporating case-based activity made it different from the traditional lecture-based teaching system. This gave students the opportunity to be more imaginative about their treatment plans. The presentations and discussions led to critical thinking and the development of collaborative strategies to address their clinical procedures. This gave them various ideas for approaching carious lesions. Problem-based learning allowed the students to develop a scenario-based treatment plan. Through discussions, the students incorporated multiple improvements. The students actively participated in peer-to-peer learning, which not only enhanced their knowledge retention and self-autonomy but also boosted their confidence. The analysis showed that students seemed to prefer interactive case-based classes and laboratory activities over their traditional teaching methodology.

The results of this study suggest that any activity that provokes critical thinking in students should be integrated into the curriculum. Such activities increase students’ preparedness for their first clinical procedures. Thus, the results support curriculum modification to include problem-based and peer-to-peer learning.

Further findings can be reinforced through the participation of more institutes to eliminate ambiguity. A future extension of this study is to evaluate the confidence level of students in the control groups. Rather than making it a compulsory session to be attended by all students by incorporating it into the curriculum, the activity session can either be optional or offered to a particular group of students. All these extensions will present a clear picture of our findings by removing any bias, thus further strengthening our outcome suggestions.

## Conclusions

The preclinical operative dentistry curriculum has been enhanced by the inclusion of case-based interactive activities. The students attended interactive classes and laboratory sessions and provided feedback at multiple stages. The results demonstrated that students' confidence and proficiency in performing cavity preparations increased significantly after engaging in case-based activities. These findings highlight the effectiveness of interactive sessions in bridging the gap between theoretical knowledge and practical skills, emphasizing the value of CBL in operative dentistry education. Further in-depth research is warranted to explore the long-term impact of CBL and its optimal implementation strategies in dental education.

## Appendices

Instructions: Please rate your confidence in the following areas using a 5-point Likert scale (1 = Strongly Disagree, 5 = Strongly Agree).

**Table 5:** Questionnaire used for assessing student confidence

Strongly Disagree	Disagree	Neutral	Agree	Strongly Agree	Questions
					I feel comfortable modifying my cavity preparation
					I mentally feel comfortable enough to design a cavity preparation
					I will not require faculty support during my first clinical procedure
					I am confident to evaluate my procedure
					I understand the basic concepts of caries removal
					I have clear concepts of cavity preparation
					I can comfortably select the appropriate required bur

## References

[1] Garg N, Garg A (2010). Textbook of Operative Dentistry. Textbook of operative dentistry: Boydell & Brewer Ltd.

[2] Chaudhary FA, Fazal A, Javaid MM, Hussain MW, Siddiqui AA, Hyder M, Alam MK (2021). Provision of endodontic treatment in dentistry amid COVID-19: a systematic review and clinical recommendations. Biomed Res Int.

[3] Overton JD (2010). What is different in operative dentistry?. Tex Dent J.

[4] Innes NP, Chu CH, Fontana M (2019). A century of change towards prevention and minimal intervention in cariology. J Dent Res.

[5] Dalli M, Çolak H, Mustafa Hamidi M (2012). Minimal intervention concept: a new paradigm for operative dentistry. J Investig Clin Dent.

[6] Ahmad P, Chaudhary FA, Asif JA (2022). Risk analysis of factors in clinical anxiety among undergraduate and postgraduate students in dentistry. Work.

[7] Barrero C, Duqum I, Petrola F (2015). Dental students' perceived preparedness to treat patients in clinic after a fixed prosthodontics course: survey results of a case study. J Dent Educ.

[8] Frese C, Wolff D, Saure D, Staehle HJ, Schulte A (2018). Psychosocial impact, perceived stress and learning effect in undergraduate dental students during transition from pre-clinical to clinical education. Eur J Dent Educ.

[9] Alkhateeb N, Salih AM, Shabila N, Al-Dabbagh A (2022). Objective structured clinical examination: challenges and opportunities from students' perspective. PLoS One.

[10] Roberts EP, Delapp JA, Florento G, Kramer RT, Brownstein SA, Stein AB (2020). Preclinical competency testing in North American dental schools and opinions about possible standardization. J Dent Educ.

[11] Al-Saud LM, Mushtaq F, Allsop MJ (2017). Feedback and motor skill acquisition using a haptic dental simulator. Eur J Dent Educ.

[12] Agarwal A, Subramaniam G, Khattak O (2023). Navigating post COVID-19 education: an investigative study on students' attitude and perception of their new normal learning environment. PeerJ.

[13] Mills D, Hammer C, Murad A (2017). Power of peers: students’ perceptions of pairing in clinical dental education. J Dent Educ.

[14] Srinivasan M, Wilkes M, Stevenson F, Nguyen T, Slavin S (2007). Comparing problem-based learning with case-based learning: effects of a major curricular shift at two institutions. Acad Med.

[15] Adamas-Rappaport WJ, Waer AL, Teeple MK (2013). A comparison of unguided vs guided case-based instruction on the surgery clerkship. J Surg Educ.

[16] Shrestha B, Subedi S, Paudel S, Subedi N, Parajuli U (2023). Impact of case based learning on teaching of undergraduate Oral Pathology course. J Nepal Health Res Counc.

[17] Khattak O, Ganji KK, Agarwal A (2023). Student perception and preferences with social media for enhanced learning in health sciences following post-COVID-19 era: a cross-sectional study. Cureus.

[18] Ali M, Han SC, Bilal HS (2018). iCBLS: an interactive case-based learning system for medical education. Int J Med Inform.

[19] Kaur G, Rehncy J, Kahal KS, Singh J, Sharma V, Matreja PS, Grewal H (2020). Case-based learning as an effective tool in teaching pharmacology to undergraduate medical students in a large group setting. J Med Educ Curric Dev.

[20] Alavi-Moghaddam M, Zeinaddini-Meymand A, Ahmadi S, Shirani A (2024). Teaching clinical reasoning to medical students: a brief report of case-based clinical reasoning approach. J Educ Health Promot.

[21] Thistlethwaite JE, Davies D, Ekeocha S (2012). The effectiveness of case-based learning in health professional education. A BEME systematic review: BEME Guide No. 23. Med Teach.

[22] Johnsen DC, Glick M (2016). The future is not ours to see, but there is always critical thinking. J Am Dent Assoc.

[23] Collares K, Opdam NJ, Peres KG, Peres MA, Horta BL, Demarco FF, Correa MB (2018). Higher experience of caries and lower income trajectory influence the quality of restorations: a multilevel analysis in a birth cohort. J Dent.

[24] Forbes HM, Syed MS, Flanagan OL (2023). The role of problem-based learning in preparing medical students to work as community service-oriented primary care physicians: a systematic literature review. Cureus.

[25] Elangovan S, Venugopalan SR, Srinivasan S, Karimbux NY, Weistroffer P, Allareddy V (2016). Integration of basic‐clinical sciences, PBL, CBL, and IPE in US dental schools’ curricula and a proposed integrated curriculum model for the future. J Dent Educ.

[26] Liu Z, Li S, Shang S, Ren X (2021). How do critical thinking ability and critical thinking disposition relate to the mental health of university students?. Front Psychol.

[27] Iqbal A, Khattak O, Chaudhary FA (2022). Caries risk assessment using the caries management by risk assessment (CAMBRA) protocol among the general population of Sakaka, Saudi Arabia—a cross-sectional study. Int J Environ Res Public Health.

[28] Stoilov M, Trebess L, Klemmer M, Stark H, Enkling N, Kraus D (2021). Comparison of digital self-assessment systems and faculty feedback for tooth preparation in a preclinical simulation. Int J Environ Res Public Health.

